# Effect of Mechanical Pretreatments on Damage Mechanisms and Fracture Toughness in CFRP/Epoxy Joints

**DOI:** 10.3390/ma14061512

**Published:** 2021-03-19

**Authors:** Chiara Morano, Ran Tao, Marco Alfano, Gilles Lubineau

**Affiliations:** 1 Department of Mechanical, Energy and Management Engineering, University of Calabria, 87036 Rende, Italy; chiara.morano@unical.it; 2 COHMAS Laboratory, Physical Sciences and Engineering Division (PSE), King Abdullah University of Science and Technology (KAUST), Thuwal 23955-6900, Saudi Arabia; ran.tao@kaust.edu.sa (R.T.); gilles.lubineau@kaust.edu.sa (G.L.); 3 Department of Mechanical and Mechatronics Engineering, University of Waterloo, 200 University Avenue West, Waterloo, ON N2L 3G1, Canada

**Keywords:** CFRP, adhesive bonding, fracture toughness, R-curve

## Abstract

Adhesive bonding of carbon-fiber-reinforced polymers (CFRPs) is a key enabling technology for the assembly of lightweight structures. Surface pretreatment is necessary to remove contaminants related to material manufacturing and ensure bond reliability. The present experimental study focuses on the effect of mechanical abrasion on the damage mechanisms and fracture toughness of CFRP/epoxy joints. The analyzed CFRP plates were provided with a thin layer of surface epoxy matrix and featured enhanced sensitivity to surface preparation. Various degrees of morphological modification and fairly controllable carbon fiber exposure were obtained using sanding with emery paper and grit-blasting with glass particles. In the sanding process, different grit sizes of SiC paper were used, while the grit blasting treatment was carried by varying the sample-to-gun distance and the number of passes. Detailed surveys of surface topography and wettability were carried out using various methods, including scanning electron microscopy (SEM), contact profilometry, and wettability measurements. Mechanical tests were performed using double cantilever beam (DCB) adhesive joints. Two surface conditions were selected for the experiments: sanded interfaces mostly made of a polymer matrix and grit-blasted interfaces featuring a significant degree of exposed carbon fibers. Despite the different topographies, the selected surfaces displayed similar wettability. Besides, the adhesive joints with sanded interfaces had a smooth fracture response (steady-state crack growth). In contrast, the exposed fibers at grit-blasted interfaces enabled large-scale bridging and a significant R-curve behavior. While it is often predicated that quality composite joints require surfaces with a high percentage of the polymer matrix, our mechanical tests show that the exposure of carbon fibers can facilitate a remarkable toughening effect. These results open up for additional interesting prospects for future works concerning toughening of composite joints in automotive and aerospace applications.

## 1. Introduction

Lightweight materials have played a significant role in product design since the inception of the aerospace industry. More recently, global trends toward emissions reduction and resource efficiency have further increased the importance of this topic [1]. Indeed, the introduction of CO${}_{2}$ emissions targets and correlated penalties has reignited the interest for lightweight materials in the automotive industries, prospecting a significant market growth [2]. Automakers are currently looking to incorporate a larger share of lightweight carbon-fiber-reinforced polymers (CFRP) within the car body to reduce greenhouse emissions and fuel consumption [3], but also to offset the weight increase associated to batteries required by electrified power trains [4].

Thermoset CFRPs have excellent properties, such as high strength, low weight, and corrosion and fatigue resistance, but the automotive industry application is limited due to the long production cycle [3]. For this reason, cured CFRP components are usually joined to the car body using a fastening method, such as riveting or bolting. The manifested limitations are the damage to the load-carrying fibers by through-holes and the generation of stress concentrations. As a result, the current pace of adoption in high-end products and critical load-bearing applications is still limited by the ability to join CFRPs efficiently [5]. Joining with structural adhesives emerged as a key-enabler of structural light-weighting because it allows for reduction of stress concentration and assembly cost and time, which are critical to the affordability of lightweight multimaterial structures [6,7,8,9].

CFRPs surfaces are primarily composed of a polymer matrix and are affected by a range of contaminants that include silicones from release agents, e.g., fluorocarbon release sprays. As contaminants are mainly embedded within the outermost surface layer of the laminates, a light removal of epoxy matrix from the adjoining interfaces has a beneficial effect on adhesion and bond reliability [10,11,12]. The most common surface preparation methods for CFRP include the use of peel-plies, corona discharge, flame, plasma, or laser treatments [13,14,15,16,17,18,19]. Peel-plies are simple to apply and provide (reproducible) surfaces that are often unsuitable for adhesive bonding, unless used in combination with another surface treatment [10]. More advanced methods require the aid of specialized equipment. For instance, corona or flame treatments usually remove weak boundary layers and oxidize the target surface, resulting in improved wetting [13]. Oxygen plasma can increase carbonyl content and etch the surface, increasing roughness, wettability, and the strength of adhesive joints [14]. Pulsed lasers at various wavelengths, including ultraviolet [15], near-infrared [16], and infrared [17,18], are also very effective and can remove embedded contaminants through photochemical or photothermal interactions, with consequent improvement of wetting and surface energy.

Recent studies indicated that specialized equipment to attain high surface free energy and roughness is not always necessary for high joint strength. Indeed, obtaining a contaminant-free matrix layer using classical grit-blasting [14] or dry end-milling [19] has enabled the successful bonding of CFRP laminates. The authors indicated that bonding must occur between the epoxy adhesive and the CFRP matrix to avoid brittle interlaminar fracture. Consequently, the surface treatments were carefully done to result in a surface with a high percentage of the polymer matrix. However, CFRP laminates are often provided with a small surface matrix layer and are quite sensitive to mechanical abrasion. Thus, it can be challenging to prevent significant exposure of carbon fibers; see for instance [15,17,18], to list a few. However, as shown in this paper, having a surface that does not feature a high percentage of the polymer matrix does not necessarily represent a weakness for a composite-bonded joint.

A common mechanism of failure occurring in monolithic CFRP is represented by fiber bridging. Such an extrinsic source of energy dissipation can provide a significant toughening effect in mode I fracture testing of composite laminates [20,21]. It might be beneficial to investigate whether a mechanical pretreatment that exposes carbon fibers can trigger large-scale bridging in adhesive-bonded composite materials. Even though several works have been reported about surface preparation of CFRP, to the best of the authors’ knowledge, such a study has not been pursued previously. The present work reports the results of a preliminary investigation that aims to fill this gap. In particular, adhesive bonding of CFRP laminates was carried out using a structural epoxy adhesive. We adopted sanding with SiC paper and grit-blasting with glass particles to afford surface preparation methods that allow a fairly controllable level of fiber exposure. We employed contact profilometry, scanning electron microscopy (SEM), and contact angle measurements to tailor surface preparation. We identified model interfaces that displayed similar wettability but featured either a light removal of the surface matrix or a significant amount of exposed carbon fibers. Mechanical tests of double cantilever beam (DCB) adhesive joints were carried out to determine the joints’ fracture toughness. In order to assess the mechanisms of failure in the course of failure, we used a high-resolution CCD camera. Obtained results open up the interesting prospect of enabling fiber bridging to maximize composite joints’ performance.

## 2. Materials and Methods

### 2.1. Substrates and Adhesive Materials

Composite substrates were prepared using carbon-fiber-reinforced polymer (CFRP) prepregs (HexPly T700/M21, Hexcel, Stamford, CT, USA), comprising a toughened epoxy matrix and nominal fiber volume fraction of about 60%. Unidirectional laminates ((0${}^{\circ }$)8) were fabricated by compression molding and a PTFE mold-release film during lay-up to generate a flat initial surface after consolidation. The mechanical properties of cured plates are reported in Table 1. Notice that the material was already used in our previous works, therefore, the detailed schedule of material fabrication [17], as well as the results of tensile tests with strain gauge measurements [22], are described elsewhere. The interlaminar fracture toughness was determined using the double cantilever beam (DCB). Typical results are reported in the Appendix A. Limited fiber bridging was observed and, as a result, a flat R-curve was recorded in repeated measurements. The steady-state (plateau) fracture toughness was equal to G${}_{Ic}$ = (0.48 ± 0.06) kJ/m${}^{2}$.

The adhesive employed for the joints’ fabrication was a toughened two-component epoxy, namely, the Araldite 420 A/B (Huntsman Advanced Materials GmbH, Basel, Switzerland). The mechanical properties are reported in Table 2. The adhesive is recommended for bonding both metals and composites and is characterized by very high peel and lap-shear strength [23,24]. The main mechanical properties are summarized in Table 1.

### 2.2. Surface Preparation

The CFRP plates were surface treated before bonding using various methods, including degreasing (DG), grit-blasting (GB), and sanding (S). The DG process was carried out using an ultrasonic bath with isopropyl alcohol for a duration of 5’. The DG step was accomplished before (and after) any of the surface preparation processes used herein. After DG, the substrates were dried at 40 ${}^{\circ }$C for 2 min before bonding. The GB treatment was carried out with the aid of an industrial sandblaster (model 0687, FERVI, Vignola, Italy), using a commercially available glass powder as the abrasive media with grain sizes in the range of 200 ÷ 300 m (i.e., 50–70 grit). The sample–gun distance (D) and the number of passes (N) were varied through the process, and we analyzed the effect on surface morphology and wettability. Instead, the blast angle was kept constant as much as possible and equal to 90${}^{\circ }$. Two levels for each variable were assessed: N = {3,6} and D = {20,50} mm. The following terminology has been used throughout the manuscript: G_NY_DZ, where G stands for the particles’ material, i.e., glass; Y represents the number of passes; and Z is the distance from the top surface. The sanding treatment was executed using silicon carbide (SiC) emery paper. We used four different grit sizes—i.e., 180, 320, 500, and 800 grit—and, based on preliminary tests, the duration was fixed to 3’, independent of the grain size used. In what follows, a specific sanding treatment is denoted by SX, where X represents the grit size.

### 2.3. Analysis of Surface Morphology and Topography

The cured plates have been imaged using a Zeiss Merlin scanning electron microscope (SEM), equipped with a Schottky field emission gun. The SEM images were acquired collecting the secondary electron (SE) signal, with the microscope working at an acceleration voltage of 5 kV, a beam current of 100 pA, and by using the in-chamber SE detector. All samples were sputter-coated with an Iridium layer of 4 nm-thickness before SEM analysis.

Surface roughness was determined using a contact profilometer (Sutronic 25, Taylor Hobson, UK). A gauge length of 4 mm was selected, while the hardware’s measurement resolution was equal to 100 μm. A minimum number of three scans was carried out to ensure redundancy and consistency of the obtained results. Measurements were done in the directions parallel (0${}^{\circ }$) and perpendicular (90${}^{\circ }$) to the orientation of the fibers, and the arithmetical mean deviation $R_{a}$ (i.e., the average deviation of all points of the roughness profile from a mean line over the evaluation length) was determined as suggested in UNI ISO 4287 [25]:

(1) $$R_{a}=\frac{1}{n}\sum_{j=1}^{n}\left|r_{j}\right|,$$

where *n* is the number of sampling points and $r_{j}$ is the height of the surface profile with respect to the reference plane. The average of these measurements ($S_{a}$) was employed to present the obtained results:

(2) $$S_{a}=\frac{R_{a,x}+R_{a,y}}{2}.$$

### 2.4. Surface Energy and Wetting Envelops

**Figure 1:**
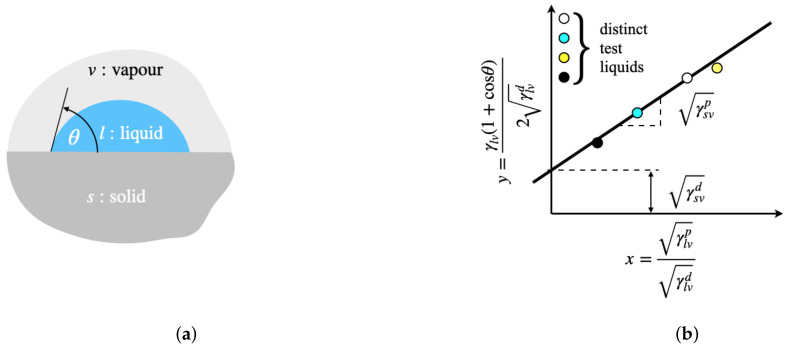
(**a**) Liquid drop resting at equilibrium on a solid surface. (**b**) Schematic Owens–Wendt plot for determining the surface free energy of a solid.

Surface free energy of the CFRP substrates was evaluated using the Owens–Wendt theory [6]. By using Young’s equation and noting that surface free energy can be subdivided into dispersive (*d*) and polar (*p*) components, the following thermodynamic equilibrium equation of a solid-liquid-vapor system (*s*-*l*-*v*) is obtained:

(3) $$\frac{\gamma_{lv}\left(cos\theta +1\right)}{2\sqrt{\gamma_{lv}^{d}}}=\sqrt{\gamma_{sv}^{p}}\frac{\sqrt{\gamma_{lv}^{p}}}{\sqrt{\gamma_{lv}^{d}}}+\sqrt{\gamma_{sv}^{d}},$$

where θ is the contact angle, which can be determined using a variety of techniques, such as the sessile drop method shown in Figure 1a. Besides, $\gamma_{lv}$ is the surface energy of the test liquid, and $\gamma_{lv}^{d}$ and $\gamma_{lv}^{p}$ are the corresponding dispersive and the polar components. Similarly, $\gamma_{sv}^{d}$ and $\gamma_{sv}^{p}$ are the dispersive and the polar component of the solid surface energy that represent the unknown that needs to be determined, since the solid surface energy is given by $\gamma_{sv}=\gamma_{sv}^{d}+\gamma_{sv}^{p}$. By recognizing that Equation (3) is in the form y=mx+q, where

(4) $$x=\frac{\sqrt{\gamma_{lv}^{p}}}{\sqrt{\gamma_{lv}^{d}}},\;y=\frac{\gamma_{lv}\left(cos\theta +1\right)}{2\sqrt{\gamma_{lv}^{d}}},$$

it is possible to determine $\gamma_{sv}$ by using different liquids with known polar ($\gamma_{lv}^{p}$) and dispersive ($\gamma_{sv}^{d}$) surface free energies and measuring the corresponding contact angle on the target surface to generate (x,y) data points. Indeed, the gradient and the intercept of the best-fit line will thus provide the polar ($\gamma_{sv}^{p}$) and dispersive ($\gamma_{sv}^{p}$) components of the solid surface energy, as schematically illustrated in Figure 1b.

In this work, the contact angle was determined through a built-in setup comprising a high-resolution camera and a micrometric syringe. Two testing liquids were selected for the analyses, i.e., distilled water and glycerol. The corresponding surface free energies are reported in Table 3.

At least three contact angle measurements were performed for each liquid, the droplet volume was controlled with a micrometric syringe and was equal to 5 μL. The obtained polar and dispersive components of the solid surface energy were then used to build $\gamma_{lv}^{d}$ versus $\gamma_{lv}^{p}$ plots, i.e., surface wetting envelope diagrams. By starting from Young’s equation, assuming full wetting (θ=0) and accounting for the additivity of components of surface energy, the $\gamma_{lv}^{d}$ - $\gamma_{lv}^{p}$ plot can be obtained from the following equation [18]:

(5) $$\gamma_{lv}^{p}-\sqrt{\gamma_{sv}^{p}\gamma_{lv}^{p}}+\gamma_{lv}^{d}-\sqrt{\gamma_{sv}^{d}\gamma_{lv}^{d}}=0.$$

For a given liquid adhesive, if the corresponding polar and dispersive components of surface free energy will provide a point enclosed within the wetting envelope, then spontaneous spreading and complete wetting are expected.

### 2.5. Fabrication of Adhesive Joints and Determination of Fracture Toughness

**Figure 2:**
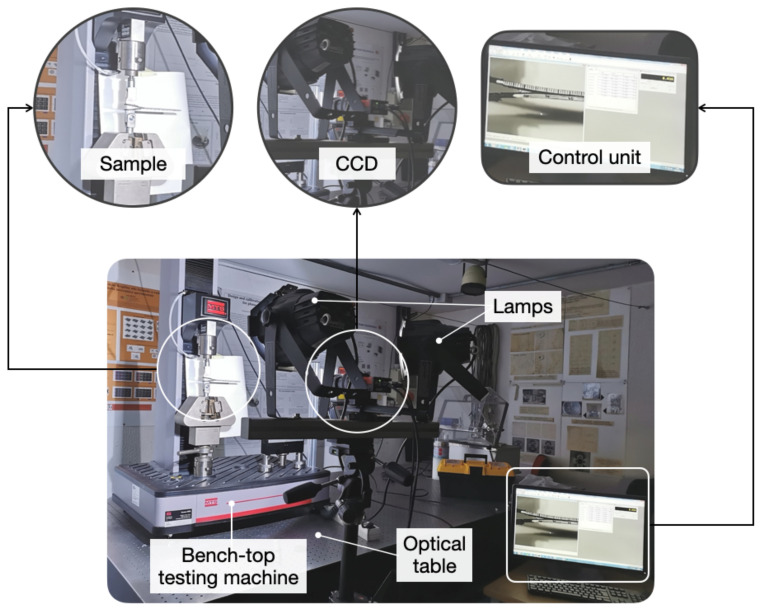
Experimental setup employed in mechanical testing of double cantilever beam (DCB) adhesive joints.

Double cantilever beam (DCB) adhesive joints were fabricated to determine the fracture toughness. The DCB is a convenient test configuration to ascertain the effect of surface contaminants or highlight deficiencies in surface pretreatments [11]. A schematic of the sample with corresponding dimensions is reported in Appendix B. Bonding was executed using the bicomponent epoxy adhesive described earlier. Adhesive curing was accomplished over a period of 24 h at 25 ${}^{\circ }$C with the aid of a climatic chamber (MTS 651, MTS Systems Corporation, Eden Prairie, MN, USA). At the end of the curing process, the samples were scrutinized below a stereoscope to assess manufacturing imperfections. Images were acquired using DFC 320 camera (Leica, Wetzlar, Germany) with dedicated proprietary software for image analysis. Displacement-controlled mechanical tests have been carried out using a bench-top electromechanical testing machine equipped with a 5-kN loading cell (MTS Criterion Model 42, MTS Systems Corporation, MN, USA). A cross-head displacement equal to 2 mm/min was used and the peel load was introduced by means of loading pins that were inserted into aluminum blocks adhesive bonded to the CFRP substrates (see Appendix B). An overview of the setup employed for the measurements is reported in Figure 2.

The crack length was measured along the edge of the specimen by using a GigE high-resolution camera (Prosilica GT) with a max resolution of 2448 × 2050 pixels, pixel dimension equal to 3.45 μm × 3.45 m, maximum frame rate of 15 fps, and a 2/3’’ CCD sensor (Sony ICX625). The camera was interfaced with commercial software (Vic-Snap, Correlated Solutions), and snapshots of the crack propagation process were acquired through an acquisition board (DAQ-STD-8D, National Instruments). A voltage proportional to the cross-head displacement was used to register an image every 0.5 mm and to correlate crack dimension with load-displacement data. Samples edges were marked every millimeter to aid crack measurement. At least four repetitions were carried out for each surface treatment. The mode-I fracture toughness ($G_{Ic}$) was determined using the procedures outlined in [26] and summarized in Appendix B.

## 3. Results and Discussion

### 3.1. Surface Morphology and Topography

**Figure 3:**
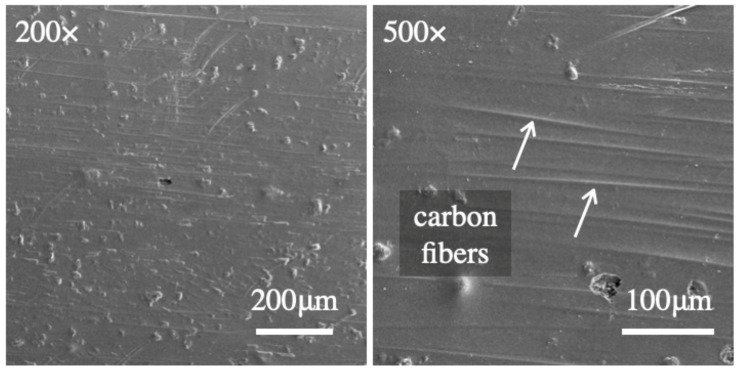
Scanning electron microscopy (SEM) images of the as-received CFRP surface after consolidation in the hot press.

The as-received (AR) material in pristine conditions is shown in the SEM images of Figure 3. Carbon fibers surfacing the outermost layer of the laminate are also highlighted. The peculiar morphology is attributed to the relatively thin surface matrix layer and the use of a PTFE mold-release film.

**Figure 4:**
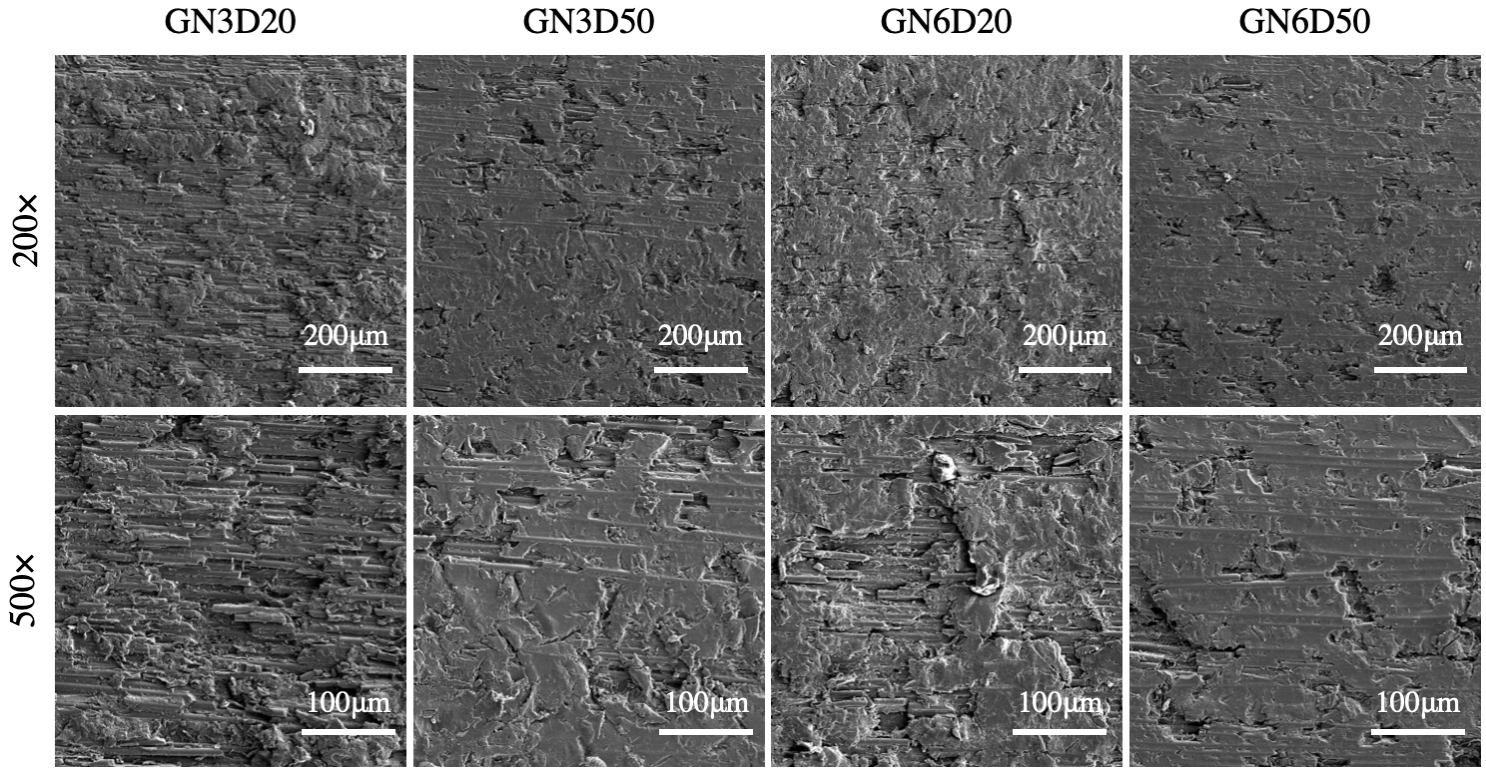
Scanning electron microscopy (SEM) images of CFRP surfaces grit-blasted using glass particles.

SEM images of grit-blasted (GB) CFRP surfaces are reported in Figure 4. The images indicate that the morphology and removal rate of the epoxy matrix are more sensitive to the gun-to-surface distance (D) rather than the number of passes (N). Indeed, by reducing D, more matrix was removed from the CFRP surface with a consequent increase of exposed carbon fibers.

**Figure 5:**
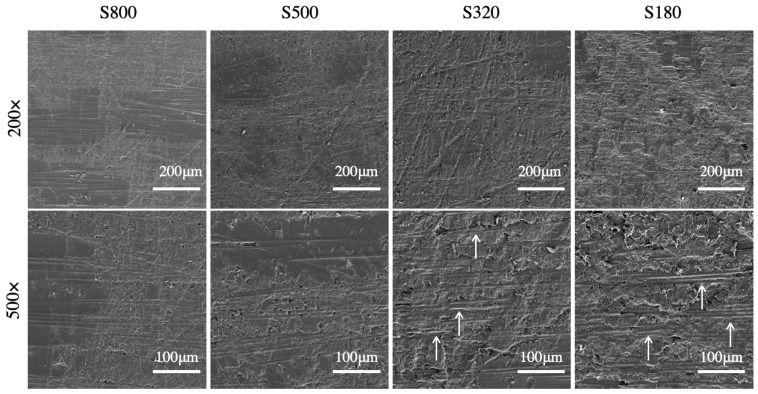
Scanning electron microscopy (SEM) images of CFRP surfaces sanded with silicon carbide papers of various grit sizes.

**Figure 6:**
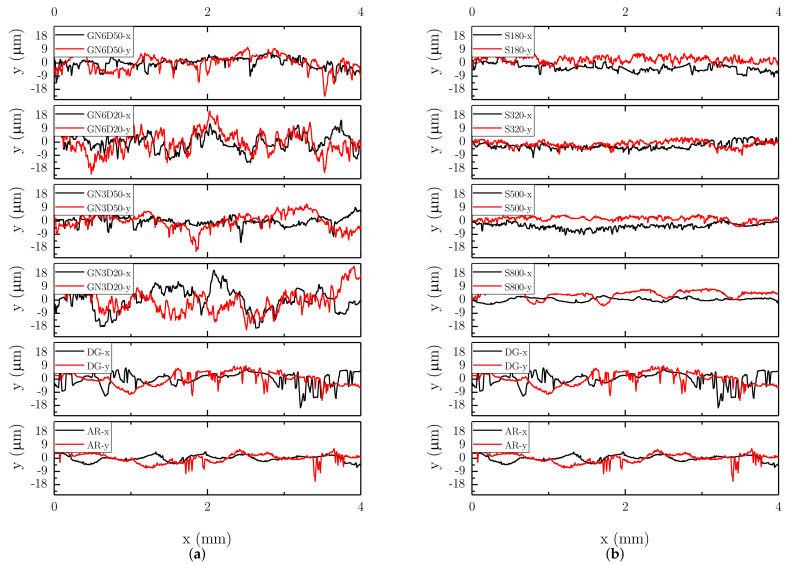
Surface profiles of CFRP plates: (**a**) grit-blasted and (**b**) sanded surfaces.

Sanding with emery paper was carried out using four distinct grit sizes and the corresponding SEM images are compared in Figure 5. The finer grits led to several surface areas with little or no changes in morphology. With 320 and 180 grits, the sanding treatment provided a more even surface modification. In a few locations across the treated area, such as those highlighted by the arrows in Figure 5, we did observe exposed carbon fibers mainly flattened by the sanding process and therefore still embedded in the epoxy. Overall, the surfaces had a high percentage of the polymer matrix. Surface profiles were extracted in the directions parallel (*x*-) and perpendicular (*y*-) to the direction of the fibers, and representative results are reported in Figure 6. The roughness-sampling-length employed for the measurements is equal to 4 mm. The baseline (AR and DG) surfaces exhibit a relatively smooth profile but with occasional peaks and valleys (i.e., millimeter range), likely due to scratches and defects induced during manufacturing and subsequent handling of the plates (see Figure 3). The surface profiles of sandblasted samples feature high-frequency microroughness. In addition, when the sample-to-gun distance was reduced (D = 20 mm), a random waviness (macroroughness) was also observed. The profile did not display such additional macroroughness when increasing the distance to D = 50 mm. The observed topography is probably due to the removal rate of epoxy matrix and random exposure of carbon fibers associated to D = 20 mm. By changing the number of passes from N = 3 to 6, we did observe significant differences in the measured profiles, as also shown in previous SEM images. As expected, the surface profile of sanded samples, which are reported in Figure 6b, are relatively smooth with respect to the baseline surfaces. The treatments further flattened surface asperities, with a significant decrease in surface roughness with respect to both baseline and grit-blasted surfaces.

**Figure 7:**
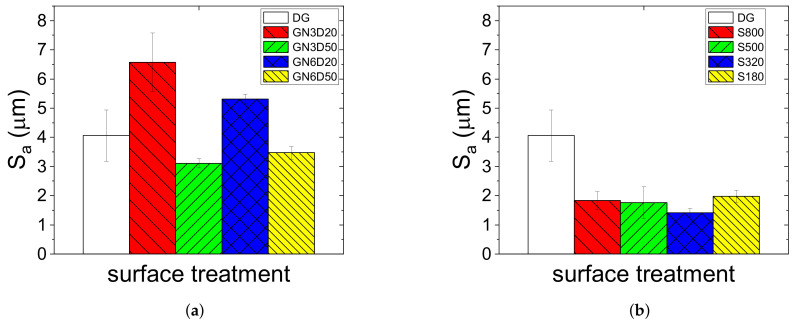
Surface roughness of CFRP plates: (**a**) grit-blasted and (**b**) sanded surfaces.

Surface roughness ($S_{a}$) was extracted from the above measurements and is reported in Figure 7. For the sandblasted surfaces, consistently with SEM and profilometry observations, $S_{a}$ was more sensitive to the gun-to-surface distance (D) rather than to the number of passes (N). However, by increasing N, reduced scatter in repeated measurements was observed. Concerning sanding, the treatment flattened surface asperities and always decreased the roughness below the values obtained with grit-blasting. The use of different emery papers did not affect $S_{a}$ to a great extent, since the values were very similar. However, as noted above, the use of 320 and 180 emery papers led to the exposure of carbon fibers.

### 3.2. Surface Energy and Wettability

**Figure 8:**
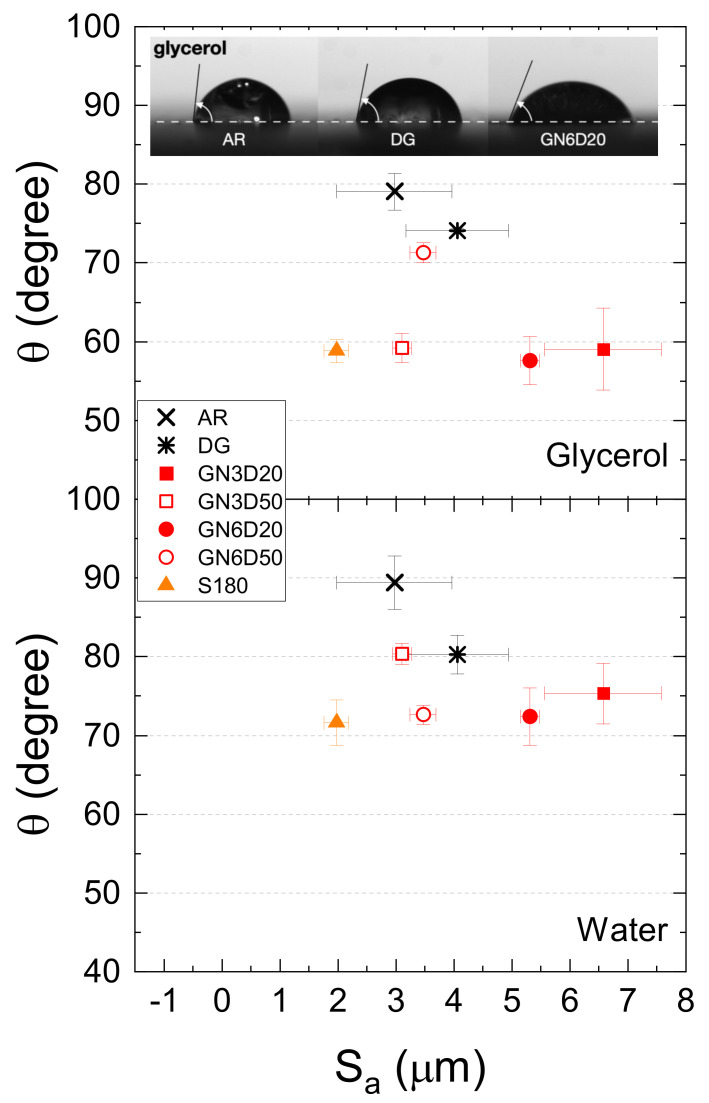
Contact angle as a function of average surface roughness obtained using glycerol and deionized water probe liquids. The insert shows drops of glycerol placed on as-received (AR), degreased (DG), and grit-blasted (GN6D20) samples.

Surface wettability was surveyed to expand the analysis and better understand the effects of the mechanical treatments. The results of contact angle measurements obtained using both glycerol and distilled water are reported in Figure 8. The reported values were determined 60 s after drop dispensing in order to spread any capillarity effect. Notice that only the surface treatment S180 was withheld for the subsequent analysis of contact angle and determination of free energy. Indeed, the CFRP samples subjected to the sanding treatments described above were preliminarily scrutinized using the water-break-free test. The samples were submerged and withdrawn rapidly from a bath of distilled water. It was noted that, except for S180, the water formed water beads (or small droplets) randomly across the surface because of improper wetting. The determined values of contact angles for the GB surfaces were consistently lower than those recorded on AR and DG. However, if compared to other combinations of processing parameters, the grit-blasting treatment GN6D20 featured a reduced scatter in repeated measurements. Despite the lower surface roughness, a similar wettability was observed for the sanded samples (S180), whose measurements were characterized by low variability.

**Figure 9:**
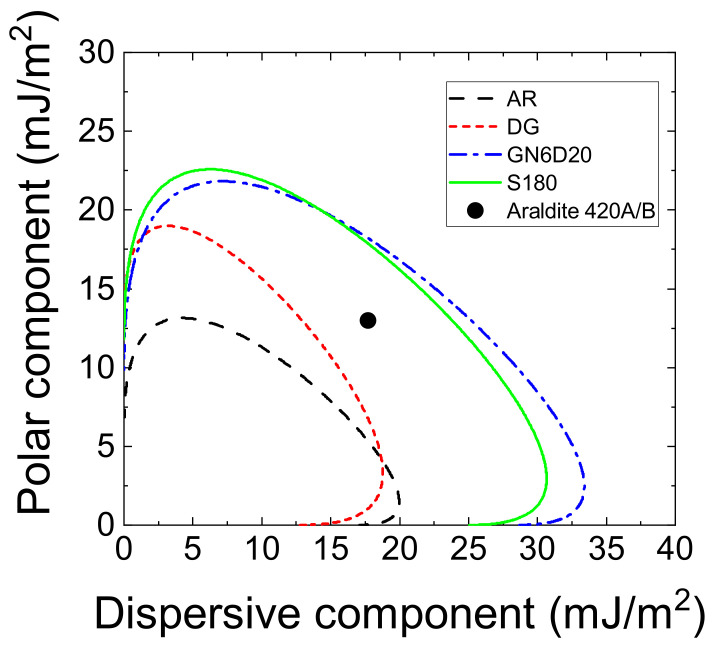
Surface wetting envelope diagrams of AR, DG, sanded (S180), and GN6D20 samples.

The solid surface energy and the wetting envelope diagrams were subsequently obtained using the methodology described earlier. The obtained diagrams for GN6D20 and S180 surfaces are compared with those related to AR and DG surfaces in Figure 9. The surface energy of the liquid adhesive, which was determined in our previous work [18], was also added to ascertain whether or not the liquid adhesive can spread on any of the given surfaces and establish the necessary intermolecular interaction needed to achieve a strong bond. Both GB and S treatments allowed for the increase in surface energy and the corresponding envelope diagrams fully embedded the liquid adhesive data point. It is interesting to note that the diagrams of GB and S surfaces are nearly identical. Since these surfaces are basically composed of a mixture of carbon fibers and epoxy matrix, it appeared that the CFRP texture had only a minor influence on the wetting behavior. Thus, the dominating factor is supposed to be surface composition. The result is consistent with the previous finding by Wetzel et al. [11] that assessed the influence of surface preparation (e.g., teflon foil, peel ply, and peel ply followed by atmospheric pressure plasma) and contamination on the fracture toughness of adhesive bonded CFRPs.

### 3.3. Double Cantilever Beam Tests

**Figure 10:**
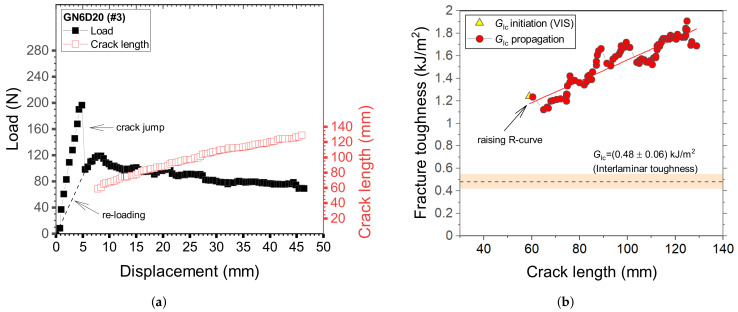
Typical results of DCB samples GN6D20 (sample #3). (**a**) Load-displacement and crack length displacement curves; (**b**) R-curve.

The fracture toughness was determined through DCB tests. At least four samples for each surface preparation method have been fabricated and tested. The load-displacement curves, as well as the crack length, were extracted during mechanical tests and the combined measurements enabled the determination of R-curves using the experimental compliance method [26]. Typical results obtained in repeated testing of DCB adhesive joints with GB interfaces are reported in Figure 10. The corresponding initiation fracture toughness, i.e., the onset of a visually recognizable crack increment as observed from the edge of the specimen (VIS), was equal to $G_{Ic}\approx$ 1.2 kJ/m${}^{2}$. As discussed later, extensive fiber bridging was observed and resulted in a significant enhancement of the propagation fracture toughness. For this reason, a steady-stated crack growth was not achieved and a raising R-curve was obtained. The peak toughness in Figure 10 is equal to ≈2.0 kJ/m${}^{2}$.

**Figure 11:**
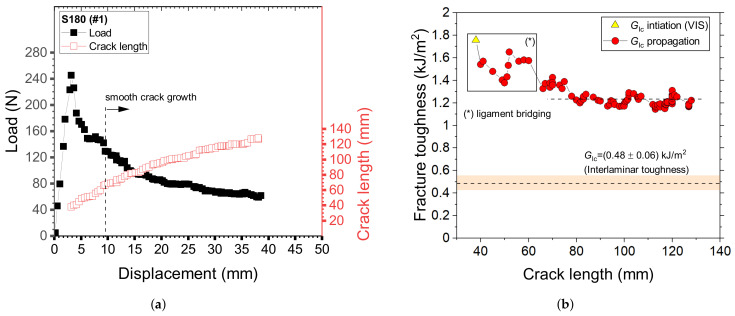
Typical results of DCB samples S180 (sample #1). (**a**) Load-displacement and crack length displacement curves; (**b**) R-curve.

Typical load-displacement curves of adhesive joints with sanded interfaces (S180) are given in Figure 11a. Crack propagation was relatively smooth and fiber bridging was not observed, with the sole exception of few outliers that have shown a very localized exposure of fiber across the interface. The smooth crack propagation process (steady-state crack growth) is reflected by the relatively constant values of fracture toughness reported in Figure 11b. However, the initial portion of the load-displacement trace as well as the R-curve were dominated by the formation of a large adhesive ligament probably stemming from the starter crack. After that, the load decay was fairly smooth and the propagation fracture toughness was relatively constant. Interestingly, as shown in Figure 10b and Figure 11b, G${}_{Ic}$ was always higher than the interlaminar toughness of the CFRP laminate.

### 3.4. Discussion

**Figure 12:**
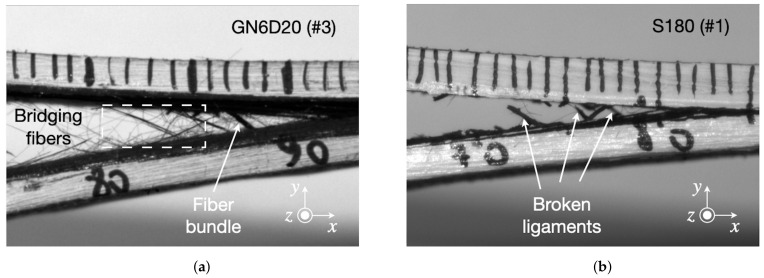
Typical mechanisms of damage recorded in (**a**) grit-blasted (GN6D20) and (**b**) sanded (S180) CFRP/epoxy joints recorded during the experiments.

During each DCB test, a region of interest surrounding the advancing crack front was recorded with the aid of a high-resolution camera to track damage in the course of fracture. The key features of the crack propagation process extracted from the images are given in Figure 12 and refer to the mechanical results shown in Figure 10 and Figure 11. For grit-blasted interfaces, crack propagation occurred in conjunction with extensive fiber bridging and provided a significant reinforcing effect. As shown in Figure 12a, single fibers and fiber bundles are created and pulled out during the fracture process. In the sanded samples, we did not observe any significant fiber bridging effect but there was competition between crack propagation at the upper and lower interfaces, as also reported in a previous work on composite joints bonded with an adhesive film [12]. The peculiar mechanism of failure has led to the formation of small adhesive ligaments bridging the crack faces. Such ligaments, which are eventually broken in the crack wake, are highlighted in Figure 12b. It is worth noting that the authors have recently demonstrated a method that enables a highly controllable formation of adhesive ligaments through tailoring interfacial adhesion [27]. The occurrence of multiple ligaments was shown to provide a significant reinforcing effect in otherwise brittle adhesive bonded composite joints.

**Figure 13:**
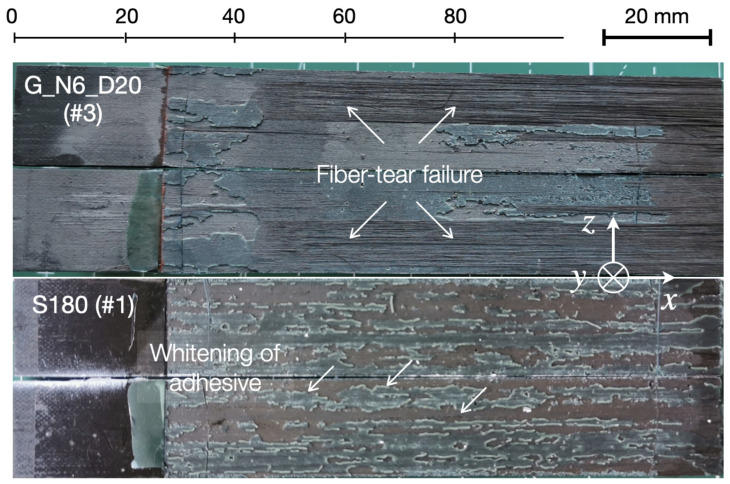
Fracture surfaces of the composite joints with grit-blasted and sanded surfaces.

The images of the fracture surfaces reported in Figure 13 provide representative information regarding the fracture mechanisms associated with GB and S samples. On the one hand, fiber-tear failure and several areas with exposed fibers are visible at the grit-blasted interfaces. On the other hand, fracture of sanded samples was characterized by significant whitening of the adhesive, which is consistent with the large plastic deformations preceding failure of the adhesive ligaments. The results highlight the importance of the composite microstructure on the mechanisms of fracture. Observations made by others at the micro- and mesoscale have reported different bridging bundle morphologies in terms of size and population in CFRP materials [20]. The mechanical treatments can lead to an interlaminar fracture involving either a few bridging fibers or multiple bundles of elongated sections (or both). It follows that the toughening effect can vary across multiple tests.

**Figure 14:**
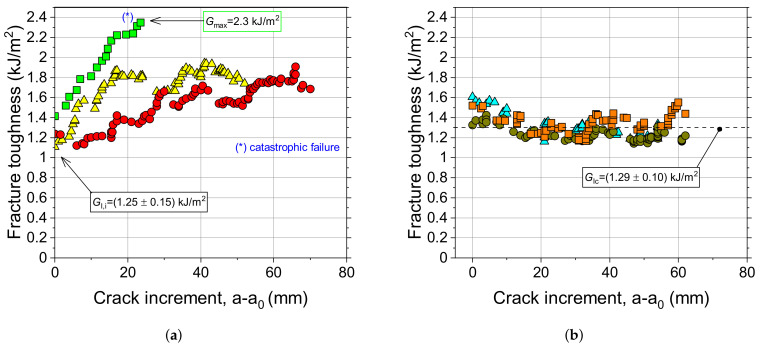
Fracture toughness versus crack increment for (**a**) grit-blasted and (**b**) sanded interfaces.

As shown in Figure 14a, the resistance curves obtained in repeated DCB tests with grit-blasted interfaces display a certain degree of variability. Although the initiation fracture toughness had a relatively consistent value, the propagation stage was characterized by a considerable degree of variation because of the different bridging extent. When large bundles accompanied fracture of the joint, the maximum toughness exceeded 2.3 kJ/m${}^{2}$, but the accumulated elastic energy within the DCB arms ultimately led to a catastrophic fracture. On the contrary, it is noted that sanded interfaces provided a consistent response across multiple tests and flat R-curves.

**Figure 15:**
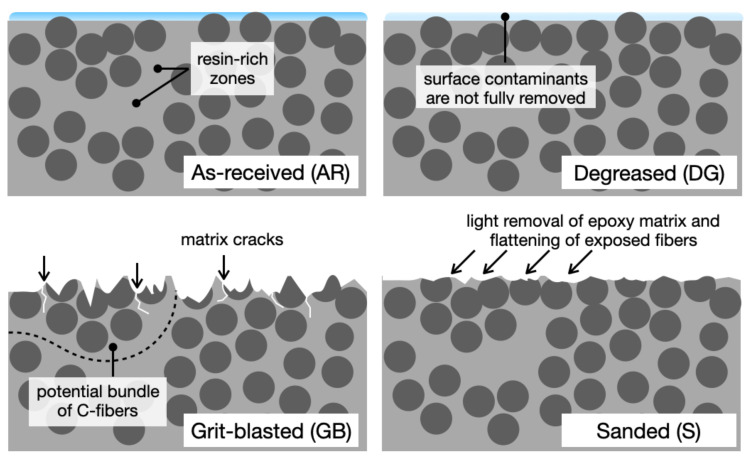
Schematic of the CFRP before and after different surface preparation methods.

The schematic of Figure 15 attempts an explanation for the observed mechanisms of failure. The initial surface (AR) is covered by contaminants that are eventually absorbed in the epoxy matrix. The use of a degreasing step (DG) is still unable to remove contaminations entirely. As our laminates feature a very thin layer of surface epoxy matrix, the GB process likely removed these contaminants. It still provided large areas with exposed loose fibers, including surface damage in the form of broken fibers and surface cracks (see Figure 4). As noted earlier, fiber dispersion within the ply and the resin-rich zone between plies plays a significant role in forming fiber bundles and on the occurrence of large-scale bridging [20]. We speculate that these surface cracks could eventually find a way through resin-rich regions of the CFRP material and promote the experimentally observed fiber bridging. Concerning sanding, the analysis of previous SEM images indicates that the treatment removed a thin outermost layer of epoxy matrix, leading to partial exposure of carbon fibers, partly flattened during the abrasion process. However, because bridging was not observed, it is concluded that the matrix/fiber interface was not compromised and the fibers were still anchored to the epoxy matrix.

The combined results pinpoint the high sensitivity of the tested material to surface preparation and that exposed fibers can significantly modify the mechanism of fracture. It is customary to attribute interlaminar fracture of composite joints to the weak resistance of laminates to peel stresses [9,28]. However, our mechanical tests show that the interlaminar fracture can provide an unexpected remarkable increase of fracture toughness by leveraging the extrinsic dissipation associated with fiber bridging as promoted thorough surface preparation.

## 4. Conclusions and Outlook

We have investigated the effects of mechanical treatments, such as sanding and grit-blasting, on the toughness of CFRP/epoxy joints. Using tailored sanding and grit-blasting treatments, we achieved a controllable level of fiber exposure across the interfaces of CFRP substrates. We identified model interfaces that displayed similar wettability but featured either light removal of the surface matrix or a significant number of exposed carbon fibers. Mechanical tests of double cantilever beam (DCB) adhesive joints revealed that surfaces mostly made of the polymer matrix provided a smooth fracture response and ensured fairly good crack propagation resistance. Besides, surface damage of CFRP and exposed fibers did not have a detrimental impact on joint resistance to crack propagation. Indeed, the associated fiber bridging promoted a beneficial R-curve behavior and a toughening effect. Remarkably, in all tests performed, the joint’s fracture toughness exceeded the interlaminar toughness of the monolithic CFRPs. In summary, this paper points out the significant effect of fiber bridging for toughening adhesively bonded joints. Our preliminary test results indicate that there is additional scope for future research opportunities. For instance, understanding how the surface of CFRPs can be engineered to ensure consistent fiber bridging levels across different tests and loading conditions would enable the fabrication of tough and damage-tolerant composite joints.

## Figures and Tables

**Table 1:** Material properties of the carbon-fiber-reinforced polymer (CFRP) plates [22].

Material	E${}_{11}$ (MPa)	E${}_{22}$ (MPa)	E${}_{33}$ (MPa)	S${}_{11}$ (MPa)	S${}_{22}$ (MPa)	$\nu_{12}$	$\nu_{21}$
CFRP	125,000	7800	7800	2138	56	0.29	0.03

**Table 2:** Material properties of the structural adhesive [23,24].

Material	E (MPa)	$S_{y}$ (MPa)	$S_{\mathrm{ut}}$ (MPa)	ν
Araldite 420 A/B	1500	27	37	0.33

**Table 3:** Polar, dispersive, and total surface free energies of the probe liquids employed for contact angle measurements.

		Surface Free Energy (mN/m)	
**Liquid**	$\gamma_{lv}^{p}$	$\gamma_{lv}^{d}$	$\gamma_{lv}$
Deionized water	51.0	22.0	73.0
Glycerol	26.4	37.0	63.4

**Figure A1:**
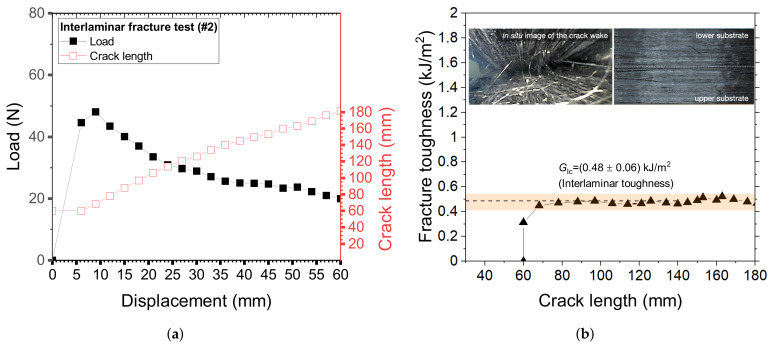
(**a**) Load-displacement and crack length displacement curves; (**b**) R-curve obtained in DCB interlaminar tests.

**Figure A2:**
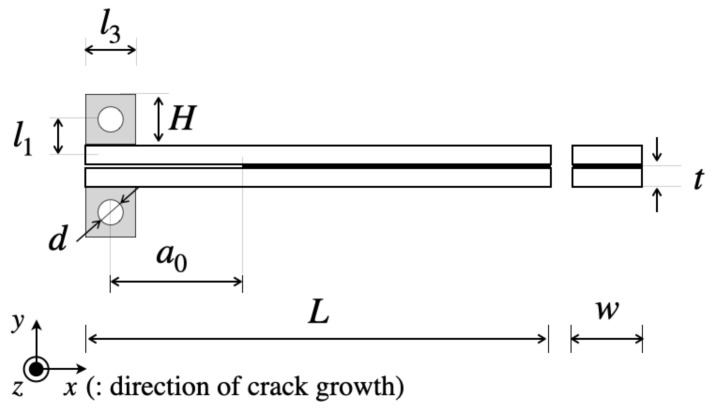
Schematic of the double cantilever beam adhesive joints comprising CFRPs adherents and bonded with an epoxy adhesive. Dimensions are not to scale.

## Data Availability

The data reported in this work are available upon request.

## Appendix A. Interlaminar Fracture Toughness of the CFRP Laminates

In this section, the results of the interlaminar fracture tests are briefly summarized. Unidirectional ([0${}^{\circ }{]}_{16}$) laminates were fabricated using carbon fiber prepregs (HexPly T700/ M21, Hexcel, Stamford, CT, USA). The fracture toughness was determined using the double cantilever beam configuration. Sample dimensions and execution of mechanical tests were based on recommendations reported in the ASTM D5528-13 [29]. The Compliance Calibration (CC) data reduction scheme was employed to determine the interlaminar toughness, and six samples were tested to ensure redundancy of the obtained results. Typical results are reported in Figure A1. In situ optical observations of the crack propagation were carried out using an industrial endoscope (CMOS Omnivision OV6946, Precision Optics Corporation Inc., MA, USA) with 400 × 400 pixels resolution. An image of the crack wake and an overview of the fractured surfaces are provided in the insert of Figure A1b. Repeated tests have shown a relatively stable crack growth and the occurrence of limited fiber bridging. This is also reflected by the flat resistance curve (R-Curve), whose plateau value reads G${}_{Ic}$ = (0.48 ± 0.06) kJ/m${}^{2}$. A detailed account of these results is reported in [30].

## Appendix B. Data Reduction Scheme for the Determination of Mode I Fracture Toughness

Double cantilever beam (DCB) adhesive joints were fabricated to determine the fracture toughness. A schematic of the sample is reported in Figure A2. A starter-crack ($a_{0}$ = 30 mm) was obtained employing an antisticking tape. Nylon wires were placed in the direction perpendicular to crack propagation (*x*-) to set the adhesive layer’s thickness to about 0.2 mm. The adjoined composites substrates had length *L* = 140 mm, width *w* = 20 mm, and thickness *t* = 2 mm. Initiation and propagation values of fracture toughness were determined using the experimental compliance method (ECM) and following the procedures described in [26]. The compliance is estimated by generating a log(*C*) versus log(*a*) diagram, where *C* and *a* are the specimen compliance and the crack length. The slope of the log(*C*)-log(*a*) curve, i.e., *m*, was employed to evaluate fracture toughness using the following equation:

(A1) $$G_{Ic}=\frac{mP\delta }{2wa}\frac{F}{N},$$

where *P* represents the load, δ is the displacement, *a* is the crack length, and *w* is the width of the specimen. Besides, *F* and *N* are corrections made to account for the effect of large displacements and for the use of loading-blocks. These corrections were made using the following equations:

(A2) $$F=1-\frac{3}{10}{\left(\frac{\delta }{a}\right)}^{2}-\frac{3}{2}\left(\frac{\delta l_{1}}{a^{2}}\right),$$

and

(A3) $$N=1-{\left(\frac{l_{2}}{a}\right)}^{2}-\frac{9}{8}\left[1-{\left(\frac{l_{2}}{a}\right)}^{2}\right]\frac{\delta l_{1}}{a^{2}}-\frac{9}{35}{\left(\frac{\delta }{a}\right)}^{2},$$

where $l_{1}$ = 16 mm distance from the center of the loading pin to the mid-plane of CFRP arm; $l_{2}$ = 20 mm is the distance between the center of the pin-hole of the load-block and its edge, measured towards the tip of the starter crack.

Notice that the thickness of the blocks was *H* = 30 mm, and the width was a match with that of the substrates. The initiation fracture toughness obtained from the mode I precrack can be determined using the following approaches: (i) NL, i.e., onset of nonlinearity on the load-displacement curve; (ii) VIS, i.e., onset of a visually recognizable crack increment as observed from the edge of the specimen; (iii) MAX/5%, i.e., the point of intersection of a straight line with the P-δ curve, with the slope of the straight line 5% higher than the initial one.
